# Fistulography-assisted endoscopic ultrasound-guided pancreatic drainage for cannulation of a non-dilated pancreatic duct in postoperative pancreaticocutaneous fistula

**DOI:** 10.1055/a-2651-9571

**Published:** 2025-09-04

**Authors:** Juan Alfonso M. Mendoza, Yu-Ting Kuo, Jirat Jiratham-Opas, Chen-Ling Peng, Thawee Ratanachu-Ek, Hsiu-Po Wang

**Affiliations:** 1 38006 Division of Endoscopy, Department of Integrated Diagnostics and Therapeutics, National Taiwan University Hospital, Taipei, Taiwan; 2 604409 Department of Surgery, Bicol Medical Center, Naga City, Philippines; 3 504608 Section of Surgical Endoscopy and Minimally Invasive Surgery, Department of Surgery, Rizal Medical Center, Pasig City, Philippines; 4 Department of Internal Medicine, National Taiwan University College of Medicine, Taipei, Taiwan; 5 37700 Department of Surgery, Hatyai Surgical Endoscopic Center, Hatyai Hospital, Songkhla, Thailand; 6 Department of Surgery, Rajavithi Hospital College of Medicine, Rangsit University, Bangkok, Thailand; 7 38005 Department of Internal Medicine, National Taiwan University Hospital, Taipei, Taiwan

Postoperative pancreatic fistula (POPF) is a serious and potentially life-threatening complication following pancreatic resection, often necessitating advanced endoscopic interventions. Endoscopic ultrasound (EUS)-guided transmural drainage and EUS-guided pancreatic drainage (EUS-PD) are established modalities for refractory POPF; however, cannulation of a narrow or nondilated pancreatic duct remains technically challenging. We present a case where fistulography was utilized to facilitate successful EUS-PD in a nondilated duct.

A 61-year-old woman with a history of central pancreatectomy for a neuroendocrine tumor presented with pancreatic juice leakage through the abdominal wall. Computed tomography revealed a fluid collection at the surgical site and a fistulous tract extending through the abdominal wall to the skin surface ( Fig. 1 ). Initial endoscopic retrograde pancreatic drainage (ERPD) failed due to severe angulation of the main pancreatic duct (MPD), allowing only a short plastic stent placement in the proximal duct ( Fig. 2 ).

**Fig. 1:**
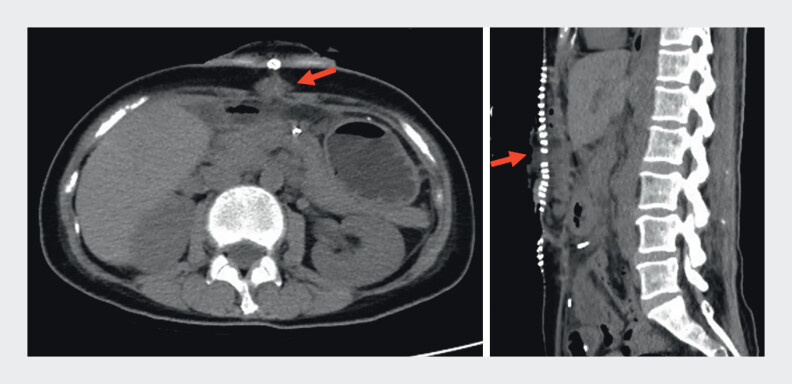
Computed tomography scan showing fluid collection at the surgical site with a tract extending to the abdominal wall (red arrow).

**Fig. 2:**
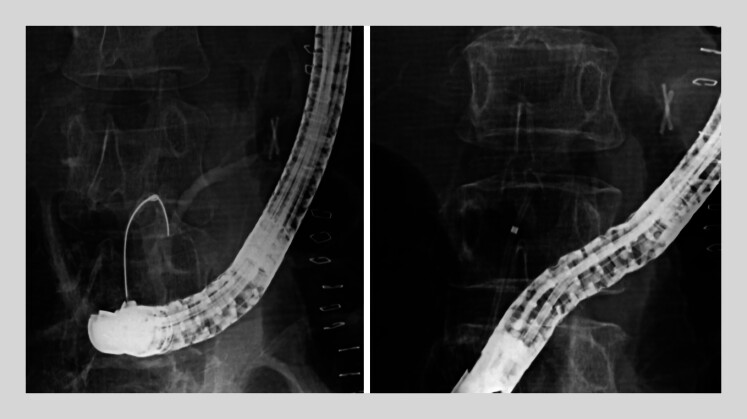
Acute angulation of the main pancreatic duct as seen on fluoroscopy during endoscopic retrograde pancreatic drainage. A plastic stent (5 Fr in diameter; 4 cm in length; Advanix Pancreatic Stent; Boston Scientific, Marlborough, Massachusetts, USA) was placed at the proximal pancreatic duct.

EUS-PD was attempted by puncturing the MPD at the body, but the guidewire could not be advanced toward the head. A second puncture at the tail was also unsuccessful due to the nondilated MPD and the patient’s rapid respiration ( Fig. 3 **a** ). To enhance ductal visualization, contrast was injected through the external fistula opening (fistulography), clearly delineating the MPD trajectory ( Fig. 3 **b** , Video 1 ).

**Fig. 3:**
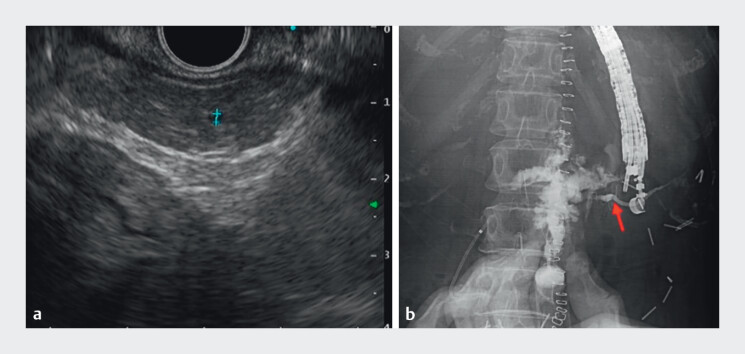
**a** Nondilated pancreatic duct on endoscopic ultrasound 1.2 mm in diameter. **b** Fistulography showing contrast extravasation, but the main pancreatic duct is now well-delineated (red arrow).

Fistulography demonstrated contrast extravasation with clear delineation of the main pancreatic duct, facilitating successful duct puncture and subsequent drainage.Video 1

Following successful duct puncture, advancement of the mechanical dilator was initially impeded by ductal angulation. Abdominal compression was applied to straighten the MPD, allowing mechanical and balloon dilation of the tract. Subsequently, a 7-Fr × 14-cm pancreaticogastrostomy stent was successfully placed ( Fig. 4 ). The pancreatic leakage progressively improved and ultimately resolved. Follow-up imaging at six months confirmed complete resolution of the fluid collection ( Fig. 5 ).

**Fig. 4:**
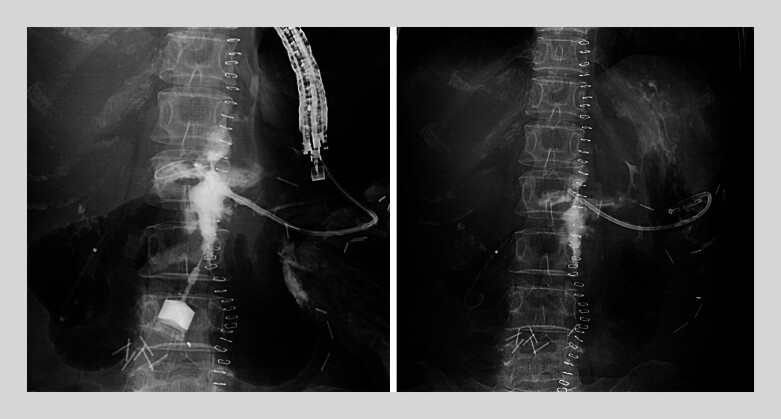
Successful placement of plastic stent (7 Fr in diameter; 14 cm in length; Through & Pass Type IT; Gadelius Medical, Tokyo, Japan).

**Fig. 5:**
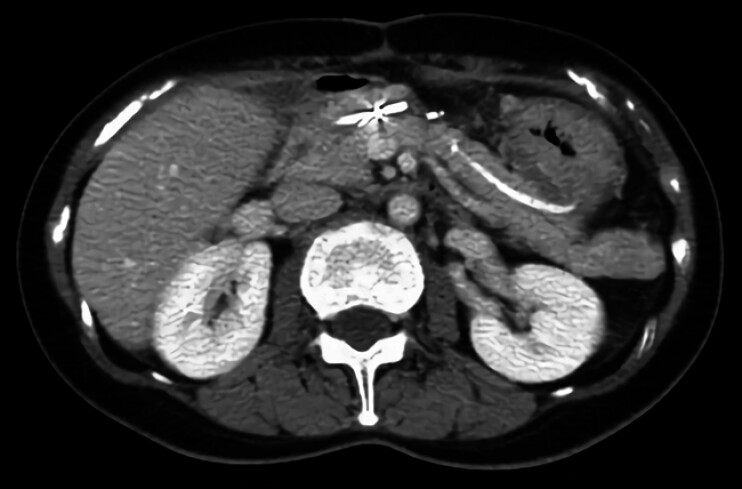
Absence of fluid collection and fistulous tract as seen on the computed tomography scan 6 months after the initial procedure.

Similar strategies utilizing adjunctive techniques to improve duct access during EUS-PD, such as balloon-assisted rendezvous, have been previously described 1 . Fistulography-assisted EUS-PD represents a promising alternative, particularly when ERPD fails 2 3 . Enhancing duct visualization with contrast injection facilitates successful cannulation and stent placement in challenging cases.

Endoscopy_UCTN_Code_TTT_1AS_2AI

## Notes

Endoscopy E-Videos https://eref.thieme.de/e-videos
*E-Videos* is an open access online section of the journal *Endoscopy* , reporting on interesting cases and new techniques in gastroenterological endoscopy. All papers include a high-quality video and are published with a Creative Commons CC-BY license. Endoscopy E-Videos qualify for HINARI discounts and waivers and eligibility is automatically checked during the submission process. We grant 100% waivers to articles whose corresponding authors are based in Group A countries and 50% waivers to those who are based in Group B countries as classified by Research4Life (see: https://www.research4life.org/access/eligibility/ ). This section has its own submission website at https://mc.manuscriptcentral.com/e-videos .
